# Virology, Epidemiology and Pathology of *Glossina* Hytrosavirus, and Its Control Prospects in Laboratory Colonies of the Tsetse Fly, *Glossina pallidipes* (Diptera; Glossinidae)

**DOI:** 10.3390/insects4030287

**Published:** 2013-07-02

**Authors:** Henry M. Kariithi, Monique M. van Oers, Just M. Vlak, Marc J. B. Vreysen, Andrew G. Parker, Adly M. M. Abd-Alla

**Affiliations:** 1 Laboratory of Virology, Wageningen University, Droevendaalsesteeg 1, Wageningen 6708 PB, The Netherlands; E-Mails: monique.vanoers@wur.nl (M.M.O.); just.vlak@wur.nl (J.M.V.); 2 Insect Pest Control Laboratories, Joint FAO/IAEA Programme of Nuclear Techniques in Food and Agriculture, International Atomic Energy Agency, Wagrammer Strasse 5, P.O. Box 100, 1400 Vienna, Austria; E-Mails: m.vreysen@iaea.org (M.J.B.V.); a.g.parker@iaea.org (A.G.P.); a.m.m.abd-alla@iaea.org (A.M.M.A.-A.); 3 Biotechnology Centre, Kenya Agricultural Research Institute, Waiyaki Way, P.O. Box 14733-00100, Nairobi, Kenya

**Keywords:** *Glossina*, *Musca*, trypanosomosis, hytrosavirus, sterile insect technique, SIT, salivary gland hypertrophy, SGH

## Abstract

The *Glossina* hytrosavirus (family *Hytrosaviridae*) is a double-stranded DNA virus with rod-shaped, enveloped virions. Its 190 kbp genome encodes 160 putative open reading frames. The virus replicates in the nucleus, and acquires a fragile envelope in the cell cytoplasm. *Glossina* hytrosavirus was first isolated from hypertrophied salivary glands of the tsetse fly, *Glossina pallidipes* Austen (Diptera; Glossinidae) collected in Kenya in 1986. A certain proportion of laboratory *G. pallidipes* flies infected by *Glossina* hytrosavirus develop hypertrophied salivary glands and midgut epithelial cells, gonadal anomalies and distorted sex-ratios associated with reduced insemination rates, fecundity and lifespan. These symptoms are rare in wild tsetse populations. In East Africa, *G. pallidipes* is one of the most important vectors of African trypanosomosis, a debilitating zoonotic disease that afflicts 37 sub-Saharan African countries. There is a large arsenal of control tactics available to manage tsetse flies and the disease they transmit. The sterile insect technique (SIT) is a robust control tactic that has shown to be effective in eradicating tsetse populations when integrated with other control tactics in an area-wide integrated approach. The SIT requires production of sterile male flies in large production facilities. To supply sufficient numbers of sterile males for the SIT component against *G. pallidipes*, strategies have to be developed that enable the management of the *Glossina* hytrosavirus in the colonies. This review provides a historic chronology of the emergence and biogeography of *Glossina* hytrosavirus, and includes researches on the infectomics (defined here as the functional and structural genomics and proteomics) and pathobiology of the virus. Standard operation procedures for viral management in tsetse mass-rearing facilities are proposed and a future outlook is sketched.

## 1. Introduction

Tsetse flies (Glossinidae: Diptera [1]) are important vectors of two debilitating diseases; the human African trypanosomosis (HAT or sleeping sickness), and African animal trypanosomosis (AAT or nagana) [2]. Tsetse flies and trypanosomoses render vast areas of agricultural land uninhabitable, especially during the rainy seasons [3]. Although over 30 species and sub-species of tsetse are described in the genus *Glossina* and most of which can transmit trypanosomoses, only 8–10 tsetse species are of medical and agricultural importance. The most important tsetse vectors are the riverine species (*G. palpalis*, *G. fuscipes*, and *G. tachinoides*) in Western and Central Africa and the savannah species (*G. morsitans*, *G. austeni* and *G. pallidipes*) in Eastern and Southern Africa [4]. Although tsetse fly fossils have been found in the 26-million-year-old shales of Florissant, Colorado, USA [5], to-date, tsetse flies are confined to Africa and in isolated populations on the Arabian Peninsula [6].

**Figure 1:**
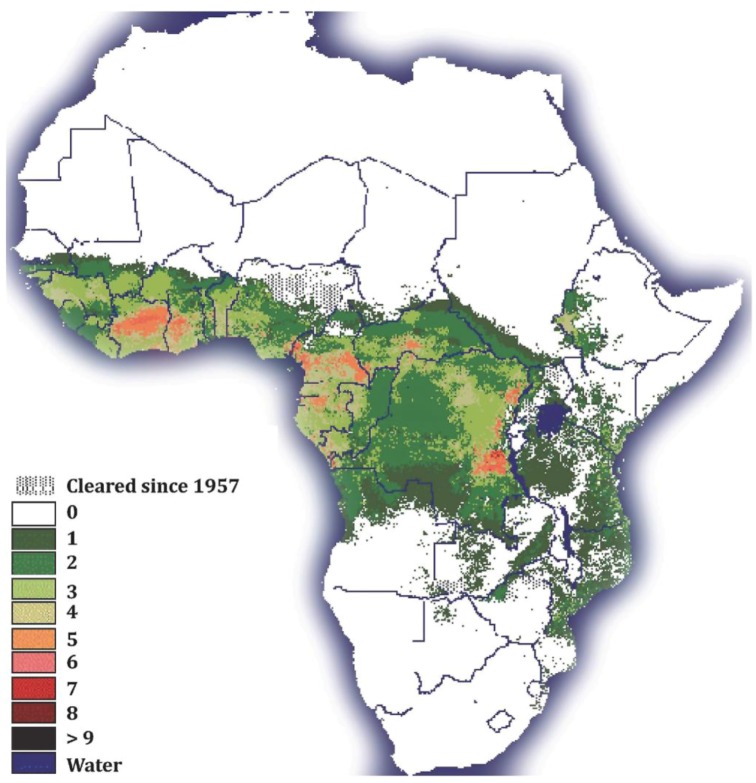
Tsetse fly distribution in sub-Saharan Africa: The Figure legend shows the numbers of different tsetse species present in sub-Saharan African countries. Note that the colors in the figure legend correspond to the colors in the figure. (Map courtesy of the FAO).

HAT is one of the most serious of the so-called “neglected tropical diseases” (NTDs [7]). NTDs are a group of chronic diseases endemic in low-income populations in Africa, Asia and the Americas [8]. Although trypanosomosis is restricted to 37 sub-Saharan African countries, its distribution extends to more than 10 million square kilometers of the African continent [9] (Figure 1).

The people at the highest risk of tsetse bites, and of contracting HAT are the rural populations that primarily depend on small-scale agriculture, fishing, animal husbandry and hunting. Resurgence and epidemics of HAT are often associated with economic decline, civil disturbance/wars, population movements and refugees [4]. The presence of tsetse and trypanosomosis is considered as one of the “roots of hunger and poverty” in sub-Saharan Africa [10]. It is estimated that approximately 90% of Africa’s livestock consists of herds in small villages [11]. This implies that maintaining healthy animals can be the difference between subsistence misery and a tolerable life for the herders and their families. The FAO estimates that ~US$ 4.75 billion worth of agricultural products are lost annually due to AAT (including ~3 million cattle deaths), and ≥100 human lives are lost daily due to HAT [12].

HAT is difficult to treat, and there are no effective vaccines are available against either AAT or HAT. None of the available trypanocidal drugs for HAT is ideal; their treatment schedules are prolonged, excruciatingly painful (often described by patients as “fire in the veins”), and require continuous hospitalization [13]. The tsetse-transmitted trypanosomes initiate their lifecycle by first colonizing the tsetse hosts’ midguts, then migrate into the ectoperitrophic space, and via the alimentary canal to the salivary glands or the mouth parts [14]. The parasites differentiate into the final mammalian-infective form (trypomastigocyte) in the tsetse salivary glands, and are then transmitted to the mammalian host by an infected tsetse bite [15]. It should however, be noted that some steps in the lifecycle of trypanosomes are group-specific. For instance, members of the *T. vivax* group only stays in the proboscis, the *T. congolense* group has a lifecycle involving the proboscis and the midguts, while only the *T. brucei* group has a cycle involving the salivary glands [15].

Without treatment, HAT can be fatal. However, fatalities of trypanosomes differ from one group to another. For instance, in West Africa *T. b. gambiensis* cause a chronic HAT that can take many years to kill a patient, while in East Africa, *T. b. rhodiensis* cause an acute HAT that can kill a patient within weeks [16,17]. The most widely used drug, melarsoprol, which was developed in 1949 [18], is lethal for up to 10% of the treated patients [19,20]. It is also important to note that, one of the biggest problems in the treatment of HAT is that the patients are usually so weak that they are more likely to die from the treatment rather than from the disease. In addition, patients need to be properly fed for several weeks to regain strength before commencement of treatments. This presents a very serious problem considering that there are hardly any available funds to properly feed or purchase drugs for HAT treatment. The available drugs for AAT are overly expensive for African peasant farmers and there are reports of increasing drug-resistance [21] and drug-counterfeiting [22]. For all the above-mentioned reasons, control of the disease vector (tsetse) is of critical importance, and likely represents the most sustainable method to manage trypanosomoses [23].

In this review, we describe the “evolution” of tsetse control methods: from traditional methods used in the early 20th Century, to modern control methods. The sterile insect technology (SIT) is considered an effective control method to eradicate tsetse populations within the frame of area-wide integrated pest management (AW-IPM) approaches [24]. Successful application of SIT to manage tsetse fly populations heavily depends on the maintenance of large tsetse colonies in mass-rearing facilities to supply the required numbers of sterile males. However, laboratory colonies of some tsetse species such as *G. pallidipes* are infected by *Glossina* hytrosavirus, a double-stranded (ds) DNA virus recently classified into the *Hytrosaviridae* family of insect viruses [25,26,27]. Virus infection often causes collapse of *G. pallidipes* colonies [28]. In this regard, we also present a historical overview of the knowledge gained about *Glossina* hytrosavirus, and consider the virology, epidemiology, pathology, and prospects for control of viral infections in laboratory colonies of *G. pallidipes*.

## 2. An Overview of Tsetse Fly Control Methods

Two main characteristics of tsetse render them suitable for eradication. Firstly, compared to other insects of medical and agricultural importance, tsetse flies have a very low reproduction rate (*k*-strategists) [29,30]. Therefore, unlike many insect vectors which produce large numbers of eggs (*r*-strategist) [30], tsetse flies have limited capacity to rebound in areas where their populations have been reduced. Secondly, tsetse flies are adapted for efficient exploitation of stable habitats offered by vertebrate nests or human dwellings with low levels of cross-breeding. This means that tsetse flies have reduced genetic variability within each vector population and therefore, have limited capacity to respond through selection pressures to various control interventions [31].

Tsetse control methods have evolved from discriminate bush clearing and wild game culling at the beginning of the 20th Century, to broadcast insecticide applications after the Second World War [32], traps, insecticide-impregnated targets [33] and live bait technologies [34]. Although these methods have been successfully used to locally reduce tsetse population sizes [29], each of these methods has limitations. Firstly, the methods do not protect the cleared areas from re-invasion by tsetse flies from residual pockets and from neighboring territories [35]. Secondly, the methods are applied in administratively-defined regions and run for an administratively-specified time [23], which mostly depend on how long external donor funds are available for the projects. Since the methods cannot be sustained beyond the time of these external donor-funded projects, the risk of the cleared areas being re-infested by tsetse flies increases. In the light of these developments, there was a need to explore other tsetse control methods.

In 1937, Knipling developed the theory of controlling insect pest population by manipulating their reproductive capacity. He likewise modeled that a target population could be eradicated when the release of sterile males was applied on an area-wide basis against an entire insect pest populations in a delineated area [36,37]. This method, commonly known as the sterile insect technique (SIT), involves large-scale production of insects in laboratory colonies in mass-rearing facilities. The male flies are then sexually-sterilized by exposure to a precise and specific dose of ionizing radiation, usually from a ^60^Co or ^137^Ce source [38,39]. The sterile males are then sequentially released into the target insect population in numbers that allows them to out-compete wild type males for wild virgin females [40]. After the virgin females mate with the sterile males, embryogenesis is arrested and consequently no viable offspring is produced. When the release of the sterile males is sustained, the size of the target insect population declines and can become extinct. The SIT is a robust control tactic that has been used very successfully against insect pests that are important in agriculture and trade. For instance, SIT was used to control Mediterranean fruit fly *Ceratitis capitata* (Diptera: Tephritidae) populations in Chile, Argentina, Mexico, Central America, South Africa, Israel *etc*. [41,42]. Lately, the SIT has also been used with great success against several lepidopteran pests such as the codling moth *Cydia pomonella* (L.) (Lepidoptera: Tortricidae) in the Okanagan Valley of Canada [43], the false codling moth *Thaumatotibia leucotreta* (Meyrick) (Lepidoptera: Tortricidae) in South Africa [44], the Australian painted apple moth *Teia anartoides* Walker (Lepidoptera: Lymantriidae) in New Zealand [45], and the pink bollworm *Pectinophora gossypiella* (Saunders) (Lepidoptera: Gelechiidae) in Texas, New Mexico, Arizona, California (US) and in Sonora and Chihuahua of northern Mexico [41,46].

The SIT played a pivotal role in the sustainable eradication of the tsetse fly *Glossina austeni* from the Unguja Island (Zanzibar) [47,48]. This program was preceded by successful applications of the technique against *G. palpalis gambiensis* and *G. tachinoides* in the Sideradougou area in Burkina Faso, and against *G. palpalis palpalis* in the Lafia area of Nigeria [49,50]. The programs in Burkina Faso and Nigeria were, however, not implemented according to AW-IPM principles and the tsetse-cleared area was re-invaded after the programs were completed. Following the area-wide eradication of *G. austeni* the island was declared tsetse-free in 1997, it still is to-date.

The success of SIT in eradicating *G. austeni* and trypanosomosis from Unguja Island inspired African Governments to call for increased efforts to manage the tsetse fly and trypanosomosis on mainland Africa. Consequently, an AW-IMP program with an SIT component was initiated in 1997 to eradicate *G. pallidipes* from a 25,000 square kilometers of under-utilized fertile land in the Southern Rift Valley of Ethiopia [51]. For this Ethiopian SIT program, a laboratory colony of *G. pallidipes* was established in 1997 at the Insect Pest Control Laboratories (IPCL) of the Joint FAO/IAEA Seibersdorf Laboratories, Austria. This colony was initiated from pupae originating from Arba Minch, Ethiopia. However, the colony collapsed in 2002. After the collapse of the colony, more pupae were shipped from Arba Minch to IPCL, Seibersdorf in an attempt to re-establish the colony. However, these attempts have been largely unsuccessful: currently, only a few of these flies (*n* = 25) are surviving, and their fecundity is very low. Further investigations revealed that the colony collapse was due to infection by a virus that caused salivary gland hypertrophy (SGH) syndrome [52,53,54]. A chronology of the emergence of the SGH syndrome and the discovery of the virus that causes the syndrome is discussed in Section 3: a summary is presented in in Table 1. 

**Table 1:** Chronological history of the discovery and distribution of hytrosaviruses.

Investigator(s)	Year	Major contribution(s)	Ref.
Whitnall	1932, 34	First published record of SGH *Glossina* spp.	[55,56]
Burtt	1945	Suggested that SGH is sex-linked	[57]
Jenni *et al.*	1973, 74, 76	Described virus particles in *G. morsitans* and *G. fuscipes fuscipes*; suggested Golgi-ER viral assembly	[58,59,60,61]
Lyon	1973	First published record of SGH in *M. equestris*	[62]
Jaenson	1978	First clear association of viral particles with SGH	[63]
Amargier *et al.*	1979	Reported SGH in *M. equestris*	[64]
Otieno *et al.*	1980	Reported SGH as common feature in wild *G. pallidipes*	[65]
Opiyo	1983	Reported poor productivity of *G. pallidipes* colony at Kenya Trypanosomosis Research Institute (KETRI), Kenya	[66]
Odindo *et al.*	1981, 83, 86	Demonstrated that viral particles are infectious *per os*; First report that *Glossina* virus has dsDNA genome	[67,68,69,70]
Jaenson	1986	First report on reduced insemination rates, fecundity and lifespan in laboratory colonies of *G. pallidipes*	[71]
Ellis *et al.*	1987	Reported SGH in Zimbabwe and Ivory Coast	[72,73]
International Atomic Energy Agency	1987, 89	Reported poor productivity of *G. pallidipes* colonies at IPCL, Seibersdorf, Austria	
Odindo	1988	Proposed *Glossina* virus as a bio-control agent	[74]
Jura *et al.*	1988, 89, 92, 93	Demonstrated transmission of *Glossina* virus after artificial infection	[75,76,77,78]
Kokwaro *et al.*	1990–1991	Cytopathology of virus particles in tsetse salivary glands	[79,80]
Shaw	1993	Reported SGH in *G. m. swyenatoni* and *G. brevipalpis*	[81]
Coler *et al.*	1993	First published record of SGH in *M. domestica*	[82]
Sang	1996–1999	Reported SGHV in tsetse milk glands, mid-gut and male accessory reproductive glands	[83,84,85,86]
International Atomic Energy Agency	2002	Collapse of an Ethiopian-derived *G. pallidipes* colony at IPCL, Seibersdorf, Austria	
Kokwaro	2006	Reported viral particles in male accessory reproductive glands of *G. m. morsitans* Westwood	[87]
Abd-Alla *et al.*; Garcia-Maruniak *et al.*	2008	*G. pallidipes* and *M. domestica* SGHVs genome sequenced	[26,27]
Abd-Alla *et al.*	2009	Establishment *Hytrosaviridae* family	[27]
Salem *et al.*	2009	Transcription analysis of *M. domestica* SGHV	[88]
Kariithi *et al.*	2010–2013	Described proteome and morphogenesis of *Glossina* SGHV	[89,90]
Prompiboon *et al.*	2010	Reported wild-wide distribution of SGHV in *M. domestica*	[91]
Luo and Zheng	2010	SGHV-like virus described in accessory gland filaments of the parasitic braconid wasp, *D. longicuadata*	[92]
Boucias *et al*.	2013	Described the role of endosymbionts on trans generational trans mission of SGHV in *G. pallidipes*	[93]
Abd-Alla *et al.*	2013	Reported successful management of *Glossina* hytrosavirus and eradication of SGH in *G. pallidipes* colonies at IPCL, Seibersdorf	[94]

## 3. A Historic Chronology of the Discovery and Biogeography of SGH

### 3.1. SGH in Dipteran Insects

In the 1930s, Whitnall reported that some *G. pallidipes* individuals collected in the Umfolosi Game Reserve, Zululand, South Africa, had grossly enlarged salivary glands [55]. In the 1970s, the enlargement of salivary glands was shown to be associated with a virus found in cytoplasmic vacuoles of the salivary gland and midgut epithelial cells of *G. fuscipes* and *G. morsitans* [56,57,58,59]. The virus was at that time described as “virus-like particles” (VLPs), morphologically resembling the VLPs previously described in *Drosophila*, mosquitoes, and nematodes [58]. The virus detected in tsetse was erroneously suggested to be an arbovirus because other hematophagous insects (mosquitoes, ticks, sandflies and gnats) had been widely known to transmit arboviruses [57].

There were several notable features that supported the suggestion of the tsetse virus as an arbovirus. For instance, the rod-shaped viral particles detected in tsetse were thought to resemble one non-typical arbovirus group, the vesicular stomatitis Indiana virus, which was found to be rod-shaped with distinctive helical symmetry [95,96]. Other notable features included the size of the viral particles, replication in the host salivary glands, and secretion of mature virions via saliva [97,98,99,100]. In addition to resemblance to arboviruses, it was also suggested that the tsetse virus was morphologically similar to baculoviruses [63].

In the 1980s and 90s, several researchers suggested that the tsetse virus is maintained in nature via transmission from the mother to her progeny and not from the male parent [71,75,83,85]. However, father-to-progeny viral transmission cannot be totally ruled out. Possibly, the lack of detectable father-to-progeny viral transmission may result from reduced transmission rates, rather than a total failure of transmission. The phenomenon of father-to-progeny virus transmission has been demonstrated in other insect viruses. For instance, in the sigma viruses (*Rhabdoviridae*), the apparent failure of father-to-progeny transmission of the sigma virus in *Drosophila* was attributed to transfer of very low viral titers to the developing embryo via the sperm [101]. In the case of sigma viruses, the virus fails to infect early germ cells and thus prevents gamete-infection, even though viral titers in the somatic cells may increase later when the fly reaches adulthood [102]. Although the *Glossina* hytrosavirus is transferred from mother to her progeny, father-to-progeny transmission cannot be totally ruled out. This revelation implies that, under undefined conditions, the virus is reactivated from a “latent” state to a symptomatic state [68]. It was proposed that the virus-induced abnormalities were contributing to the natural regulation of tsetse populations in the field [68]. This suggestion appears to be supported by reports that related viruses such as baculoviruses regulate the population dynamics of their insect hosts in a density-dependent manner, some of which do not induce disease symptoms [103].

SGH symptoms have been reported in two other dipteran insects: in adult populations of the narcissus bulb fly, *Merodon equestris* (Diptera; Syrphidae) [62,64], and in infections in the house fly, *Musca domestica* L. (Diptera; Muscidae) collected at a dairy in Florida, USA [82]. Notably, the main disease symptoms (SGH) of the virus in the bulb fly and in the house fly were similar to those observed in the tsetse [62]. To date, there has been no further research performed on the *Merodon* virus. Although the *Glossina* and *Musca* viruses share pathological impacts on their respective hosts such as seasonal fluctuations in the incidence of SGH symptoms [68,91] and suppression of reproductive fitness in the host [75,82,85,86], the two hytrosaviruses differ in several aspects. For instance, unlike the tsetse virus, the house fly virus appears not to be maintained in nature by mother-to-progeny transmission [104,105]. Notably, whereas there is no documented evidence that the tsetse virus has potential to infect heterologous hosts, the house fly virus can infect, reduce egg production and the lifespan in the stable fly, *Stomoxys calcitrans* (Diptera; Muscidae) [106], but it did not induce expression of SGH symptoms in *S. calcitrans* [106].

### 3.2. Possible Hytrosaviruses in other Insect Species

Recently, a virus was fortuitously detected in hypertrophied accessory gland filaments (AGFs) of the parasitic wasp, *Diachasmimorpha longicuadata* (Ashmead) (Hymenoptera; Braconidae) [92]. The virus, presumed to cause the hypertrophy of the AGFs had ultra-structural features similar to those causing SGH in dipteran hosts [92]. The wasp virus was detected in a *D. longicuadata* that originated from Hawaii and released in Thailand and subsequently introduced to southern China as a bio-control agent for the oriental fruit fly, *Bactrocera dorsalis* (Hendel) (Diptera; Tephritidae) [92]. Inferring from other insect-viral systems, this discovery of the virus in the wasp may be significant. *B. dorsalis* is distributed throughout Southeast Asia and the Pacific, and is considered to be one of the most prominent agricultural pests in this part of the globe [107]. *D. longicuadata* is known to introduce *D. longicuadata* entomopoxvirus (DlEPV) into Caribbean fruit fly *Anastrepha suspensa* (Diptera: Tephritidae) during oviposition [108]. DlEPV, the first reported endosymbiotic entomopoxvirus, protects the wasp’s eggs by inhibiting encapsulation by the host’s hemocytes [109]. Consequently, one might argue that, similar to the DlEPV, the virus detected in *D. longicuadata* may potentially be an endosymbiont to the wasp. Unfortunately, similar to the *Merodon* virus, the virus detected in the AGFs of *D. longicuadata* has not been further investigated.

## 4. Pathology of Hytrosaviruses

**Figure 2:**
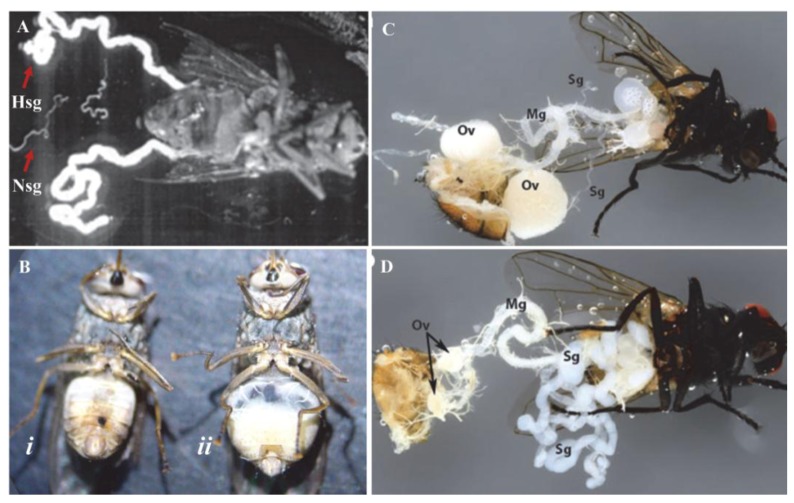
Pathology of hytrosaviruses: (**A**) Normal (Nsg) and hypertrophied (Hsg) salivary glands dissected from *G. pallidipes*. It should be noted that the pair of Nsg are dissected from a different fly for comparison with the Hsg. Notice that the glands exhibiting salivary gland hypertrophy (SGH) symptoms are enlarged ≥5 times the size of normal glands; (**B**) Male *G. pallidipes* with asymptomatic (***i***) and symptomatic (***ii***) salivary glands; (**C**) Female *M. domestica* with healthy and (**D**) hypertrophied salivary glands showing lack of ovarian development in the virus-infected fly (**D**). Abbreviations: Mg, midgut; Ov, ovary; Sg, salivary gland. (Figure sources: Panel A [28]; panel B [110]; panels C and D [111]; used with permission).

As indicated in Section 3, dipteran adults infected by hytrosaviruses can exhibit overt SGH symptoms. Flies with SGH are recognizable to the naked eye by the swollen opaque-white appearance of the abdomen, and sac-like course textures [55,60] (Figure 2).

Notably, whereas the SGH syndrome in *Glossina* mainly results from cellular hypertrophy (enlargement), SGH in *Musca* reflects a combined effect of both cellular and nuclear hypertrophy. Pathological effects of the *Glossina* hytrosavirus in colonized *G. pallidipes* were first documented the early 1980s. In 1979, a *G. pallidipes* colony was initiated at the Kenya Trypanosomosis Research Institute (KETRI) using flies caught from the Kibwezi forest, Kenya. This colony collapsed within two years of its establishment due to poor productivity [66]. Investigations were done to identify possible causes of the poor performance and eventual collapse of the colony. The study parameters included dissections of female spermathecae (to check insemination rates) and male testes (to check presence of motile spermatozoa as an indicator of male efficiency) [66]. The results demonstrated deterioration of insemination in females, aspermia, reversed ovariole development, and distortion of sex ratio. Other hytrosavirus-induced collapses of *G. pallidipes* colonies have been reported over the last two decades [28,52]. The colony collapses are a direct consequence of testicular degeneration (in males) and ovarian abnormalities (in females).

One of the outstanding questions to be answered is: why does the *Glossina* hytrosavirus cause such serious problems in colonized *G. pallidipes*? It should be noted that such serious impacts are yet to be reported in other tsetse species. Further, other insect cultures (e.g., *Drosophila*) do not seem to experience such virus-induced problems. Although this question needs more research, inferences can be made from other viral-insect systems to understand the case of *Glossina* hytrosavirus pathology. It is important to keep in mind that insect viruses hardly “walk alone”: viruses co-infect (simultaneously) their hosts with other micro-organisms such as the vertically-transmitted bacterial endosymbionts [112,113,114]. Population dynamics and roles of endosymbionts on viral infections have been documented in the infection of *Drosophila* by sigma viruses. Boucias *et al.*, have recently suggested that the pathological effects of *Glossina* hytrosavirus on colonized *G. pallidipes* flies are modulated by interplay between the virus and tsetse endosymbionts [93]. Specifically, the researchers highlighted the absence of *Wolbachia* in the laboratory stock of *G. pallidipes* that was used in the study. Although *Glossina* hytrosavirus has been reported in other *Wolbachia-*harboring tsetse species [115], so far, no harmful impacts have been reported on the virus-infected tsetse fly species that harbor *Wolbachia*. It has been reported that the levels of PCR-detectable *Glossina* hytrosavirus in *G. fuscipes fuscipes* populations are influenced by the tsetse genotype, and inversely correlate with *Wolbachia* prevalence [116]. Notably, *Wolbachia* is also absent from some of the major economically and medically important species of mosquitoes such as *Anopheles* spp. [117]. Studies have also demonstrated that while none of the *Wolbachia*-infected *Ae. aegypti* tested positive for Dengue virus after oral infection, 30%–100% of *Wolbachia*-free mosquitoes were virus-infected [118]. It can therefore, be hypothesized that the absence of *Wolbachia* in the *G. pallidipes* colonies explains the severe negative impact of *Glossina* hytrosavirus on large-scale colonies. Considering the shared intracellular locations of *Glossina* hytrosavirus and *Wolbachia*, this endosymbiont may influence the outcome of viral infection in tsetse hosts.

## 5. Virology of the Hytrosaviruses

### 5.1. Genomics of Hytrosaviruses

**Figure 3:**
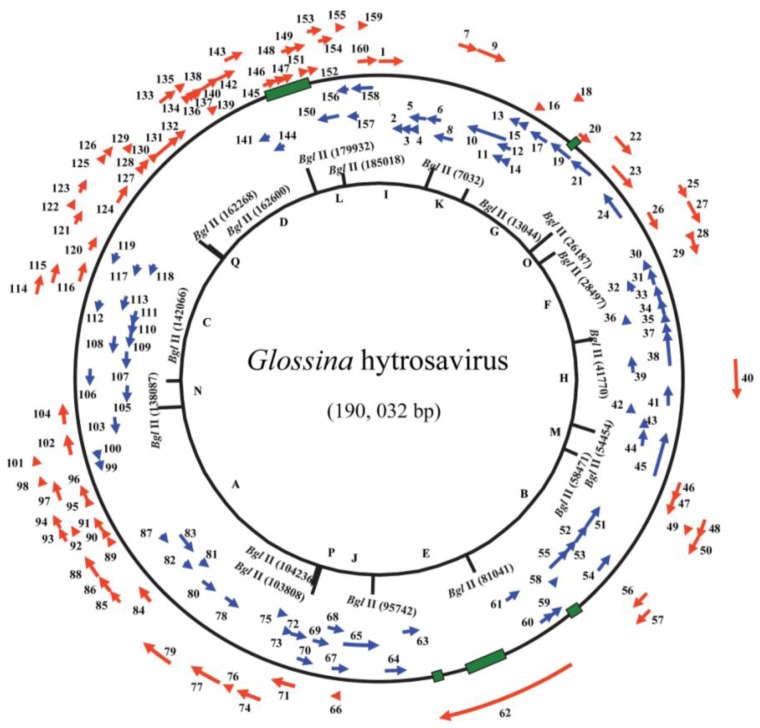
Circular representation of the *Glossina* hytrosavirus genome: Arrows indicate positions and directions of transcription for the putative open reading frames (ORFs). The ORF numbers and putative genes are shown. The alphabetical numbers represent restriction fragments generated by *Bgl*II enzyme during the electrophoretic profiling of the viral genome.

To understand the pathobiology of *Glossina* hytrosavirus, the virus was purified from hypertrophied salivary glands dissected from *G. pallidipes* flies originating from Tororo, Uganda in 1975. This colony was initially maintained at Leiden University, The Netherlands, and subsequently transferred to IPCL, Seibersdorf, Austria in 1982. Twenty-six years later, the genome of the virus was fully sequenced (NC_010356.1) [26]. The 190 kbp-long viral genome is a circular dsDNA molecule [26] (Figure 3), and represents an entirely new group of insect viruses. A total of 322 non-overlapping open reading frames (ORFs) were identified, of which 160 ORFs were presumed to encode viral proteins. One hundred thirteen (70.6%) of these ORFs did not match to any of the sequences available in the various databases [26]. Thirty-seven ORFs (23.1%) were homologues to genes of other viruses, while ten (6.3%) were homologues to non-viral/cellular genes. Most notable of the *Glossina* virus ORFs was the presence of homologues to five of the so-called *per os* infectivity factor genes (*pifs*) (*p74*, *pif-1*, *pif-2*, *pif-3*, and *odv-e66*) encoded by baculoviruses, nudiviruses and whispoviruses [119]. Other notable homologies included homologues to sixteen (entomo-) poxvirus genes, three iridovirus and nimavirus genes each, two ascovirus genes and one herpesvirus gene. Homologues to cellular genes included chitinase, DNA helicases, thymidylate synthases, and several bacterial endosymbionts genes [26]. Approximately 3% of the *Glossina* virus genome is characterized by an inverted repeat (*ir*) sequence, and fourteen direct repeat sequences (*drs*) composed of 51–246 bp.

The *Musca* virus genome has also been sequenced [120]. The viral genome is a ~124 kbp-long circular dsDNA molecule [120], with a total of 108 putative ORFs. Similar to the *Glossina* virus, over 70% (76/108) of the putative *Musca* virus ORFs had no significant homologies to proteins available in various databases. Notably, the two viruses share at least eight homologs of baculovirus core genes, including homologues to the *pif* genes, and to cellular genes [120]. Eighteen tandem *drs* were distributed throughout the viral genome [120]. The size of the repeated sequences ranged from 149 bp (dr15) to only 9 bp long (*dr*18), and the number of copies of the *drs* ranged from 1.9 to 7.4, making the total sizes of the *drs* from 30 bp–380 bp-long. The G + C content of *Musca* virus is 43.5%, a ratio similar to that found in several nudiviruses: *Heliothis zea* nudivirus-1 (HzNV-1) and *Oryctes rhinoceros* nudivirus (OrNV), but significantly higher than that of *Gryllus bimaculatus* nudivirus (GbNV; 28%) and the *Glossina* virus (28%) [121,122,123]. 

The above-mentioned hytrosavirus gene homologies to other insect viral genes have several implications. Firstly, the conserved nature of the *pifs* among all the baculoviruses that have so far been sequenced [124], and their importance in early oral infection [125], may reflect a common ancestry or similar modes of transmission and infection of the hytrosaviruses, baculoviruses, nudiviruses and nimaviruses. Secondly, the presence of repeat regions in the genomes of the these hytrosaviruses possibly reflects a common mode of transcriptional regulation and DNA replication [126]. Thirdly, the presence of the ORFs potentially encoding orthologues of cellular proteins may be advantageous for the viral infection. For instance, it has been suggested that large DNA viruses gain independence of the transcription and replication machineries of their hosts by encoding their own functional cellular orthologues of cellular proteins, mostly enzymes [127]. It remains to be ascertained whether the *Glossina* and *Musca* hytrosavirus ORFs do encode functional cellular orthologues, and what their functional roles are.

### 5.2. Classification and Phylogeny of Hytrosaviruses

Initially, the *Glossina* virus could not be assigned to any of the families of insect DNA viruses described at that time [69]. Accumulated data from various studies on the viral pathobiology singled-out signature of hytrosaviruses are shown in Table 2. Notably, in contrast to many other invertebrate large dsDNA viruses such as baculoviruses and entomopoxviruses, virions of hytrosavirus are not “packaged” in occlusion bodies. Based on these characteristics, the virus was proposed to be accommodated into a new virus family, *Hytrosaviridae* [27], a name derived from “*Hy*per*tro*phia *s*ialo*a*denitis”, the Greek word for “salivary gland inflammation”. The *Glossina* virus is now a member of the newly-established *Hytrosaviridae* family, genus *Glossinavirus*, as the species *Glossina hytrosavirus* [27,128]. Similarly, based on the shared characteristics between *Glossina* and *Musca* viruses, the *Musca* virus is the second member of *Hytrosaviridae* family, but accommodated in the genus *Muscavirus*, and species *Musca hytrosavirus*. Hereafter, the *Glossina* and *Musca* hytrosaviruses are abbreviated as GpSGHV and MdSGHV, respectively.

**Table 2:** Signature characteristics of hytrosaviruses: The table summarizes the principal biological, structural and molecular characteristics of the GpSGHV and MdSGHV. Table modified from [129]; used with permission.

Key characteristics		GpSGHV	MdSGHV	Ref.
**Biological**	Replication site(s)	Salivary glands, milk glands	Salivary glands	[75,82,83,104,130]
	Infection phenotype	Symptomatic; asymptomatic	symptomatic	[28,93]
	Symptoms besides SGH	Male/female gonadal abnormities	Under-developed ovaries	[75,82,93,105]
	Vertical (trans-generational) transmission	Milk glands, trans-ovarian	No evid ence available to-date	[52,75,82,93,105]
	Horizontal transmission	Oral (salivary) secretions	Oral (salivary) secretions and excreta	[25,52,131]
	Sterilizing agent	Male and female infertility	Female infertility	[86,105]
	Impact on host behavior	Impaired feeding	Mating disruption	[84,105,132]
	Morphogenesis	Cytoplasmic envelopment, egress by disintegration or rapture of the plasma membrane	Cytoplasmic, egress via budding on the plasma membrane	[93,133,134]
**Structural**	Virion size	50 × 1000 nm	65 × 550 nm	[120,134]
	Ultra-structure	Nucleocapsid, tegument, envelop, outer surface projections	Nucleocapsid, envelop	[89]
	Virion topography	Helical surface projections	Braided, bead-like surface	[89,120]
**Molecular**	Genome size	190,032 bp	124,279 bp	[26,120]
	G + C content (%)	28	44	[26,120]
	No. of RFs	160	108	[26,120]
	Shared ORFs between GpSGHV and MdSGHV	41	37	[90,128]
	ORFs homologs in other large dsDNA viruses	Nudivirus (11), whispovirus (4), baculovirus (12)	Nudivirus (17), whispovirus (6), baculovirus (12)	[26,120]

Phylogenetic analysis of GpSGHV and MdSGHV based on the DNA polymerase gene (*dna pol*), which is present in all large dsDNA viruses, does not cluster these hytrosaviruses with other insect dsDNA viruses [26,129]. Instead, the *dna pol* of GpSGHV and MdSGHV clusters more closely to that of herpesviruses and other viruses with linear dsDNA genomes. On the other hand, the alignments free method using whole proteome phylogenetic analyses of dsDNA viruses shows close association of the hytrosaviruses and nimaviruses (specifically the white spot syndrome virus; WSSV) [135,136,137]. Despite the apparent ambiguities, these and other phylogenetic methods, such as super tree and super matrix methods [138,139], support the notion of a common ancestry of GpSGHV and MdSGHV with baculoviruses, nudiviruses and nimaviruses [124,139]. The hytrosaviruses share 12 out of the 31 baculovirus core genes identified to date [139], and are therefore, more distantly-related to baculoviruses than for instance the nudiviruses: Nudiviruses share 20 of baculovirus core genes [124].

### 5.3. Proteomics and Interactomics of Hytrosaviruses

The presence of protein-coding regions or ORFs in a viral genome does not necessarily imply the presence of functional proteins. For instance, frame shift mutations caused by “indels” (insertions/deletions) in viral genomes can alter the structure and functions of the encoded proteins resulting in decrease or complete loss in protein expression [140]. Further, some genes may not have functional promoters.

Proteomics can be used to validate presence of virally-encoded proteins. Although the repertoire of biological processes controlled by viral gene products is complex [141], proteomic analysis of the GpSGHV made it possible to reconstruct a dynamic view of the viral infection process [89,90]. A combined approach of proteomics and electron microscopy revealed that the GpSGHV virion consists of four morphologically distinct structures: nucleocapsid, tegument, envelope, and helical surface projections. Almost 50% of the virally encoded proteins were found to reside within the viral tegument, reflecting potential roles in cytoplasmic trafficking of the virions during infection [89]. A limited proteome of the MdSGHV proteome is also available [133]. Although GpSGHV and MdSGHV share more than thirty protein homologues [90], the ultra-structural features of the MdSGHV differ from GpSGHV, but have not been studied in detail. GpSGHV also incorporates cellular proteins derived from infected cells, which may significantly contribute to the viral morphogenesis [89]. It is not yet known whether MdSGHV incorporates any host-derived cellular proteins into its mature virion, but it is very likely.

Equally important to the repertoire of viral proteins composing the viral proteome are the interactions encrypted by both the viral and host genomes. Analyses of the secretome of GpSGHV-infected *G. pallidipes* revealed that up to twenty of the secreted host proteins potentially interact with at least twenty-five viral proteins [142]. GpSGHV alters the protein expression patterns in the host salivary glands, and ≥40% of both virally-encoded and host proteins are specifically expressed in the symptomatic flies but not in asymptomatic flies [142]. The GpSGHV-host are important because, in the wild tsetse populations, the viral infections are primarily asymptomatic, whereas in a laboratory setting, at least for *G. pallidipes* colonies, the response to GpSGHV can be highly symptomatic. This suggests that in the wild tsetse populations, GpSGHV is mainly in covert (latent or persistent) infection state, and the virus is only reactivated to symptomatic state under certain specific ecological and/or environmental conditions. It is not clear which viral and/or host proteins may be expressed during viral latency. However, Lee *et al*. reported that latency can be caused by the infection of *Spodoptera frugiperda* (Sf) cells with *Autographa californica* multiple nucleopolyhedrovirus (AcMNPV) that lack the anti-apoptosis gene *p*35 [143]. Notably, even during the latency, infectious AcMNPV particles were continuously produced. Under the conditions used by these investigators, it appears that there is a balance between protein expression and the apoptotic pathway. Whereas GpSGHV does not have any homologue of any known anti-apoptotic gene, MdSGHV contains a homologue of *Melanoplus sanguinipes* entomopoxvirus anti-apoptotic gene, *iap* [120]. It should be noted that one of the major differences between GpSGHV and MdSGHV is that, MdSGHV does not have asymptomatic infection: instead, the virus induces symptomatic infection within 48 h post oral infection [144]. In addition, unlike GpSGHV, there is no evidence of vertical MdSGHV transmission. Since the issue of latency in hytrosaviruses is currently being debated among the Hytrosavirus Study Group of the ICTV, we briefly address it in Section 5.4 below.

### 5.4. Latency of Hytrosaviruses

Latency of insect virus infections is probably widespread. Latency can be defined as a viral infection that does not produce visible disease symptoms (e.g., SGH in the case of GpSGHV), but the virus may be transmitted, either vertically or horizontally [145]. This phenomenon raises a number of questions, particularly in the case of hytrosaviruses, which is the subject of this review.

The first question about latency is: is latency advantageous to the virus? Possibly, latency is an evolutionary viral strategy to utilize cues from the host and/or environment as opportunities for dispersal and transmission. In 1992, Fuxa *et al*. argued that in the adults of the armyworm *S frugiperda* (Lepidoptera: Noctuidae) latency may provide dispersal and reproductive advantages to *S frugiperda* nucleopolyhedrovirus (SfNPV) [145]. As the insect host metamorphoses to adulthood, vertical transmission becomes epizootiologically more important than horizontal transmission. The virus, therefore, takes advantage of the changes in the infected cells’ quality to produce nonlethal, vertically transferable virions. In 1993, Hughes *et al*. described a latent infection in a laboratory colony of the cabbage moth, *Mamestra brassicae* (Lepidoptera: Noctuidae) that was vertically transferred from one generation to another without disease symptoms [146]. Latency may also be influenced by virus-microbiome interplay as has been recently suggested [93]. Latency may also be stress-induced: for instance by crowding, food shortage, and superinfection in some baculoviruses [147,148]. Latency may also be connected to the antiviral response mechanisms of the hosts. Recently, it has been found that invertebrate DNA viruses, such as baculoviruses, iridoviruses and nudiviruses, provoke a specific RNA interference (RNAi) or microRNA response, which may prevent premature death of the host by preventing viral over-replication [149,150,151]. In case of the tsetse fly, there may be a further interaction between GpSGHV, the microbiome and the trypanosome parasite, and this tripartite interaction may be tailored to prevent trypanosome transmission [152].

The second question on viral latency is: how is viral latency established? One strategy in which viruses are vertically transmitted is by integrating into the host genome. For instance, the dsDNA polydnaviruses (PDVs) are vertically transmitted as proviruses stably integrated into the genomes of the parasitic wasps, ichneumonid (Hymenoptera; Ichneumonoidae) and braconid (Hymenoptera; Braconidae) [153]. Another strategy of vertical viral transmission is as infectious virions, such as the *gypsy* elements in *D. melanogaster*, which are transmitted as infectious particles from mother to progeny via oocytes [154]. Other insect viruses are maternally transmitted to the progeny flies, but also rely on horizontal transmission to be sustained in the host populations. An example of a virus utilizing this strategy is the DNA *Leptopilina boulardi* filamentous virus (LbFV) that infects the solitary parasitic wasp *L. boulardi* (Hymenoptera: Figitidae). When both an LbFV-infected and a non-infected female wasp lay eggs in *Drosophila* larvae during oviposition, LbFV is horizontally transmitted to the offspring of the non-infected wasp parent [155]. Consequently, the daughters of the non-infected females acquire the super-parasitizing phenotype (conferred by LbFV-infection) on emergence, with a 55% probability [156]. Other insect-infecting viruses, such as dengue virus (DENV) and GpSGHV, are primarily horizontally transmitted, but are also maternally transmitted [111,157]. Whether or not hytrosaviruses do integrate into the host genome is an issue currently under investigation.

### 5.5. Replication, Morphology and Morphogenesis of Hytrosaviruses

**Figure 4:**
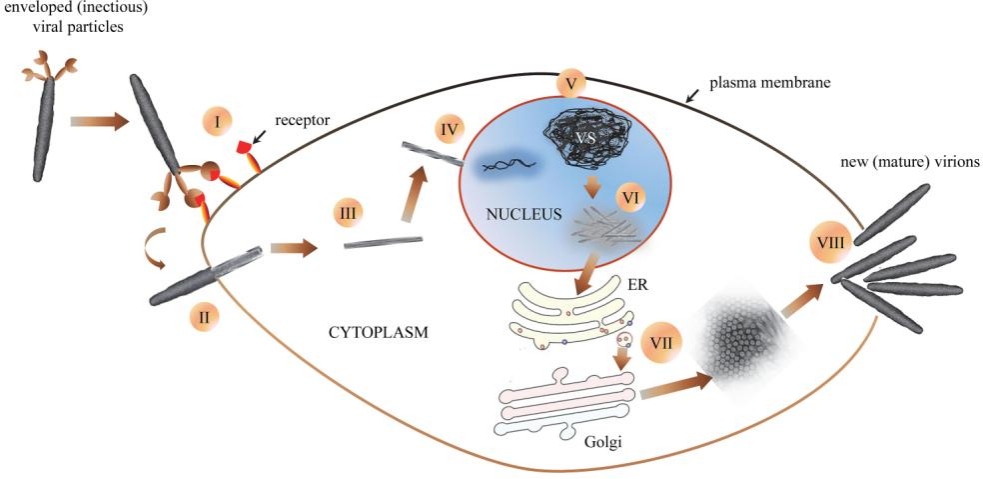
Schematic (hypothetical) representation of GpSGHV morphogenesis: (**I**) an enveloped (infectious) viral particle binds to receptors on susceptible host cell. (**II**) Once bound, the virus is uncoated as it enters the host cell (**III**). Cytoplasmic trafficking of the viral nucleocapsid to the nucleus ensues (**IV**), followed by disassembly of viral nucleocapsids by partial degradation of capsid and tegument proteins and release of viral DNA into the host cell nucleus. (**V**) Once in the nucleus, the virus induces formation of virogenic stroma (VS), where viral nuclear replication occurs. (**VI**) After packaging of nascent viral DNA into capsids, nucleocapsids are assembled, after which they egress into the cell cytoplasm. (**VII**) The entire envelopment of nascent nucleocapsids is orchestrated in the cytoplasm, possibly via the ER-Golgi system. (**VIII**) Egress of the new mature virions from the infected cell possibly occurs via rupture or disintegration of the plasma membranes.

The GpSGHV replicates in the nucleus of host cells, where it induces formation of virogenic stroma (chromatin-like network of electron-dense filaments) [89]. After nuclear assembly the GpSGHV progeny nucleocapsids translocate to the cytoplasm where the envelopment is orchestrated, possibly via the ER-Golgi system, based on residual ER-Golgi proteins in the virion proteome. The possibility of the ER-Golgi assembly of the tsetse virus was proposed in the 1970s [59]. Based on the GpSGHV pathobiological data obtained to date (see summary in Table 1), cytoplasmic assembly of the virus particles induces cellular damage that possibly culminates into disintegration of the cell plasma membrane as the mature virions egress from the infected cell [89]. The virions are continuously shed via the saliva into the blood meals during membrane feeding in *G. pallidipes* colonies, and are infectious *per os* to healthy flies [52,142]. A remarkable difference between GpSGHV and MdSGHV is that the MdSGHV virions migrate to, and bud out of the plasma membrane of infected cells [133,134]. Unlike GpSGHV, MdSGHV does not have surface projections. Based on the available data, an infection model for GpSGHV can be hypothesized (Figure 4).

## 6. Epidemiology of Hytrosaviruses

### 6.1. Prevalence and Ecogeography of Hytrosaviruses

Early epidemiological surveys established that the prevalence of SGH symptoms in wild tsetse populations depends on the geographical location, seasonality, the distribution, age and species tsetse flies [65,68,72,73], and that the prevalence was generally low (0.9–15% in Kenya [65,68], 0.4–2% in Zimbabwe [72], 0.3–4.5% in Burkina Faso [73]). However, these surveys were based on the occurrence of SGH symptoms by fly dissections, which did not take into account that the majority of GpSGHV infections are asymptomatic [110]. Recently, PCR-based surveys were performed on *G. pallidipes* samples that were randomly collected from eleven geographical locations in six countries of eastern and central Africa [54]. The PCR-based surveys showed that an average of 34% of *G. pallidipes* samples were GpSGHV-infected: viral prevalence ranged from 2% to 100% [54]. Moreover, GpSGHV diversity was noted to be low. Twenty-three different viral haplotypes occurred in the same geographical locations. The GpSGHV haplotypes distribution patterns were somewhat confused: some of the viral haplotypes occurred only in certain geographical locations compared to others.

Unlike tsetse flies, house flies are among the most widely distributed insects, found in all inhabited areas of the world [158]. The global distribution of house flies allowed isolation of MdSGHV was isolated from the fly samples collected from various geographical locations around the globe *i.e.*, from North America, Europe, Asia, the Caribbean, and the south-western Pacific [91,159]. The prevalence of MdSGHV infections in the field house fly populations can peak at ~30% at certain geographical sites, with a typical prevalence range of 0.5–10% at any given sampling period [104]. Further, the frequencies of MdSGHV infections positively correlate with the house fly population densities [104]. To date, it is unknown what MdSGHV haplotypes circulate among house fly populations. However, ecogeographic and behavioral attributes of the tsetse and house flies may influence selection pressure of GpSGHV and MdSGHV. Unlike in the case of GpSGHV, the opportunity to expose MdSGHV to the external environmental conditions such as UV radiation has probably led to the higher genetic variability of MdSGHV compared to the GpSGHV [54,91].

### 6.2. Transmission Dynamics of GpSGHV in the Laboratory Fly Colonies

**Figure 5:**
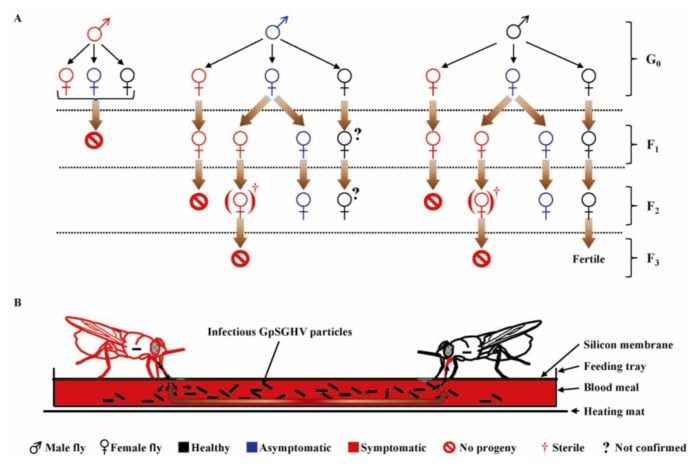
A model of dynamics of vertical (**A**) and horizontal (**B**) GpSGHV transmission in laboratory colonies of *G. pallidipes.* The laboratory colony flies in the colony may either be “healthy” (PCR-negative; shown in black), asymptomatic—(in blue), or symptomatic—(in red). (**A**) Some symptomatic (SGH-positive) females do produce F_1_ progeny flies and never F_2_ (regardless of the status of the males that inseminate the females); (**B**) During membrane feeding, virus particles released by symptomatic flies via saliva into blood meals are infectious to other healthy flies. G_0_, F_1_ and F_2_ represent parental, 1st and 2nd fly generations, respectively. (?) Represent progeny flies with unknown infection status. (Figure modified from [53]; used with permission).

Studies have revealed that GpSGHV-infected flies typically exhibit two infection phenotypes: a chronic non-debilitating asymptomatic state and an acute symptomatic state that causes reproductive dysfunction and colony collapse [93]. Various crosses were made between “healthy” (asymptomatic; PCR-negative) and symptomatic flies [52], whose outcomes are summarized in an infection model shown in Figure 5 [53]. Three key features of GpSGHV infection dynamics in laboratory colonies of *G. pallidipes* should be noted. Firstly, all males with SGH symptoms are fully sterile. Secondly, females with SGH symptoms do produce progeny flies; most (if not all) of these progeny flies exhibit SGH symptoms. Thirdly, asymptomatic females produce a small proportion of SGH-positive progeny flies; if such progeny flies are female, the F_2_ generation is sterile. The large numbers of virus particles released via saliva into the blood meals by the GpSGHV-infected flies during the *in vitro* membrane feeding (Figure 5B) are a source of *per os* transmission of GpSGHV to healthy flies [52].

Taken together, the following implications are apparent. Firstly, as hypothesized by Jaenson in 1986 [71], survival of *G. pallidipes* colonies may depend on the infection status of the mothers: all F_2_ progeny flies produced by symptomatic mothers are sterile. Males with SGH symptoms are fully sterile and do release GpSGHV particles into blood via saliva during membrane feeding, and thus have a considerable impact on colony survival. Secondly, the observations that symptomatic flies die-off from the colony early in their life-span, and that asymptomatic flies have a relatively higher efficiency in salivary virus secretion compared to the symptomatic flies (Kariithi, unpublished) imply that asymptomatic flies may have more influence on the dynamics of GpSGHV transmission in the colonies compared to symptomatic flies [93]. The higher efficiency in viral transmission by asymptomatic compared to symptomatic flies results from the latter having impaired salivary secretions [89]. These studies revealed two noteworthy features. Firstly, when *G. pallidipes* flies were orally-infected with GpSGHV, the viral titers increased with the number of contaminated blood meals taken by the flies [52]. In marked contrast to tsetse flies, house flies are resistant to oral MdSGHV infections after 24 h post emergence (hpe) [91]. It can therefore be concluded that it is not the feeding history, but rather the age (hpe) of the tsetse fly when it takes the first (infective) meal that determines the fly’s susceptibility to viral infection [160]. Notably, the proportion of orally-infected *G. pallidipes* flies that secreted viral particles via saliva during the feeding events increased from 20% in the first blood meal to 100% in the seventh meal [52]. So, what hypotheses can explain why the efficiency of virus secretion increased with number of blood meals?

The peritrophic membrane (PM) of the intestinal tissue constitutes a physical barrier for viral diffusion into the salivary glands via the hemolymph. There is still conflicting information about the function of the PM in insects. Possible functions include protection of the stomach epithelium [161], ultrafiltration [162] and preventing the entry of microbes [163]. It has been suggested that the PM may limit the vectorial capacity of some hematophagous insects such as the sandflies [164]. In other insects such as lepidopterans, the PM is an age-dependent major barrier to baculovirus infection *per os* [165]. Further, Blackburn *et al.*, [166] suggested that the PM is an impenetrable barrier for the diffusion of Leishmania or Trypanosoma parasites at certain stages after the blood meal. In mosquito-transmitted viruses, only 0.1% of Zika virus was shown to penetrate to the hemocoel of *Aedes aegypti* because of the barrier effect of the PM [167]. Subsequently, it has been suggested that the PM is a possible limiting factor for virus susceptibility in mosquitoes [168]. Likewise, the barrier function of the PM might have prevented GpSGHV from reaching the salivary glands of experimentally-infected *G. pallidipes* for at least 48 h-post-blood meal. Thereafter, the PM starts to decay and its disintegration may be completed a few days after, depending on availability of blood meal as has been suggested in sandflies [169]. It has been demonstrated that viruses enhance infection by disintegration of the PM [170]. There is strong evidence that GpSGHV virions contain chitinase [89,90], probably to facilitate the virus to penetrate the PM, which is essentially composed of chitin and proteins (mucins) [171]. This function has been demonstrated in the infection of mosquitoes by Plasmodium. The *Plasmodium* secretes chitinase to penetrate the PM in *Ae. aegypti* (inhibition of chitinase blocks transmission of Plasmodium) [172]. The important physiological functions of the PM suggest that it can be considered a significant structural target for the control of GpSGHV in the laboratory colonies of *G. pallidipes*. The potency of anti-chitinase antibodies to block transmission of pathogens has been demonstrated with the creation of Plasmodium-refractory mosquitoes [173]. A similar approach could be applied in GpSGHV management in *G. pallidipes* colonies.

Orally-infected *G. pallidipes* flies secrete infectious GpSGHV particles via saliva [52]. In contrast, when micro-injected with GpSGHV suspension, the flies did not secrete any detectable viral particles during the same duration of membrane feeding events [93]. Notably, expression of SGH symptoms in the F_1_ progeny produced by virus-injected mothers increased exponentially from 0–3% in the first larviposition cycle to 100% in the fourth cycle [93]. So, what hypotheses explain the apparent delayed expression of SGH symptoms in the F_1_ progeny produced in the early larviposition cycles? Possibly, if oocyte GpSGHV infection did not occur early in oogenesis, the developing egg membrane (chorion) might protect eggs from infection. Since the mothers ovipositioning later were blood-fed a few days post-infection, their ovaries probably became infected before egg maturation. It should be noted that in tsetse flies, larviposition is influenced by blood feeding, and is correlated to the digestion stages of the blood meal [174]. Unlike in the oral-infection, the virus injections were done on flies that had been fed (non-teneral). This implies that for the virus-injected flies, the egg chorion, possibly already formed at the time when the virus can reach the reproductive organs. This could be a further barrier for GpSGHV infection of eggs and thus of the F_1_ progeny. 

Taken together, the invasion of *G. pallidipes* ovaries by GpSGHV could take place only during the later larviposition cycles. Since maturation of eggs begins shortly after blood meal ingestion [174], it is unlikely that GpSGHV in the blood meal would have sufficient time to infect the midgut, disseminate through the hemocoel to the ovaries and infect the developing oocytes immediately following the initial virus-contaminated blood meals. Therefore, trans-ovarial transmission of GpSGHV would not occur until the subsequent larviposition cycles after the infectious blood meal advance.

## 7. Potential of Hytrosaviruses as Bio-Pesticides

Many insect-pathogenic viruses such as baculoviruses are effective bio-control agents against insect pests [175,176]. The potential of GpSGHV as a male sterility factor in tsetse control was first proposed in 1988 [74]. After micro-injection of the virus into laboratory-bred *G. pallidipes*, infection was observed in 13.3% and 30.0% of treated male and female parent insects, respectively. The prevalence of SGH in the F_1_ progeny adults was much higher than in the parents (80% in males and 58.3% females). Whereas all infected females were fertile, all infected males had SGH syndrome and were sterile. Maternal larviposition, F_1_ pupae weight, and F_1_ pupae incubation periods were normal regardless of treatments. Two other studies reported that, although GpSGHV-infected males had reduced reproductive potential [86], such males did not lose their mating efficiency [77]. Although all the studies were performed under laboratory conditions, which probably differ from field conditions, it was hypothesized that *Glossina* hytrosavirus could be applied as a tsetse bio-control: the sterile male parents would compete with normal wild males in mating, and the fertile but infected females would transmit the virus trans-ovarially to subsequent generations, since such females produce only infected progeny [71], where males are sterile from eclosion. However, as discussed in previous sections, males with SGH are not sexually competitive. Further, SGH females produce sterile progeny flies, so the line quickly dies out.

Application of GpSGHV as a bio-pesticide for tsetse control however, is technically challenging for several reasons. Firstly, recent findings show that neither micro-injection nor *per os* infection of the virus in *G. pallidipes* result in SGH syndrome in the same (parental) generation, rather, the syndrome is only detectable in the third (~65%) and fourth (~100%) larviposition cycles of the F_1_ generation [93]. So, the effects of the viral infection are not immediately apparent. Secondly, high SGH prevalence in *G. pallidipes* colonies reduces the mating propensity and competitiveness of males thus affecting the stability and performance of tsetse colonies [133]. The colony instability would hinder production of large numbers of infected insects. Thirdly, *in vitro* mass production of GpSGHV for field applications is currently impossible due to limitations such as the absence of a cell culture system permissive to the virus. Currently, the only alternative method to multiply GpSGHV is by intra hemocoelic injection of the virus into colony flies, or by feeding the flies with virus-contaminated blood meals. Even if it were feasible to multiply GpSGHV by the injection method, it is a laborious process, and would require maintenance of huge number of flies. Fourthly, there is no available evidence for horizontal transmission of GpSGHV through contact between flies, mating, or fecal contamination, thus limiting the modes of how the virus would be dispersed in the field. Finally, GpSGHV does not produce occlusion bodies, as for instance baculoviruses do to achieve prolonged stability in the environment [177]. Besides, there is evidence that GpSGHV is highly unstable outside of the host [89], with more than 80% of purified virus suspension losing infectivity after 3 days at 4 °C [53], possibly due to the loss of the fragile viral envelope. Formulation of GpSGHV suspensions to allow retention of the viral infectivity under both laboratory and field conditions appears insurmountable at the moment. Therefore, the use of the GpSGHV as tsetse bio-control agent appears currently impractical.

## 8. Strategies to Control GpSGHV in Laboratory Colonies of *G. pallidipes*

### 8.1. Immune-Intervention Strategies

In view of the current understanding of GpSGHV epidemiology under laboratory conditions, two potential strategies to manage the viral infections in the colonies were considered. The first strategy was to reduce or inhibit horizontal GpSGHV transmission by either (a) modifying the feeding system currently used in the colony rearing [94], or (b) neutralizing the virus released via saliva into blood meals during membrane feeding by supplementing the blood meals with GpSGHV-specific antibodies, and/or (c) targeting virus ligands on host midgut cells with a phage display peptide library to block viral attachment to the midgut receptors [178,179]. The second strategy was to reduce or inhibit viral replication by: (d) oral administration of antiviral drugs to inhibit viral DNA polymerase [180], and (e) silencing essential GpSGHV genes by bacterially-expressed double-stranded RNA (dsRNA)-RNAi. Preliminary data so far obtained from the immune-intervention strategies, *i.e.*, neutralizing antibodies, phage display technologies, and RNAi have shown potential to significantly reduce GpSGHV infections. Currently, these immune-intervention strategies are being optimized.

### 8.2. Modifications of *in vitro* Membrane Feeding Regime

The objective of various research reviewed in this paper was the development of a cost-effective strategy to manage the GpSGHV infections in *G. pallidipes* colonies. This was finally achieved mainly by modification of the *in vitro* membrane feeding regime routinely practiced in tsetse mass-rearing facilities. Since the membrane feeding favored horizontal viral transmission, it was conceptualized that modifications of the colony maintenance protocol(s) would significantly reduce GpSGHV transmission. The routine *in vitro* feeding regime involved feeding up to 10 sets of fly cages in succession [181], thus significantly augmenting horizontal GpSGHV transmission in the colonies. In the modified feeding regime, each fly-holding cage was provided with fresh blood at each meal to prevent flies from picking up any virus deposited via saliva into the during feeding of earlier fly cages [94]. Within 2 years of implementation of the modified feeding regime, GpSGHV loads in the fly colonies were significantly reduced and maintained at levels not detrimental to the survival and productivity of the colonies. More importantly, the SGH syndrome that causes colony collapse [28], was completely eliminated [94]. Additionally, the modified feeding regime is applicable in combination with other management strategies, for instance oral administration of the antiviral drugs in an integrated approach [180], or supplementing blood meals with GpSGHV-specific antibodies. The implications of the successful GpSGHV management in *G. pallidipes* colonies are discussed in sub-Section 8.3.

### 8.3. Implications of Successful Control of GpSGHV Infections in Colonized G. pallidipes

The membrane feeding system used in the tsetse mass-production facility located in Tanga, Tanzania greatly contributed to the production and release of ~8.5 million sterile males between 1994 to 1997, resulting in a crash of the *G. austeni* population in the Unguja Island [47]. However, attempts to mass-produce *G. pallidipes* for the SIT component of AW-IMP programs on mainland Africa were faced with difficulties because this tsetse species is susceptible to infections by GpSGHV. The virus has caused collapse of *G. pallidipes* colonies that were initiated in Ethiopia and Seibersdorf, Austria, and thus prevented full implementation of the release component. Now that strategies are available to successfully manage GpSGHV infections that can result in complete elimination of SGH from *G. pallidipes* colonies, production of sufficient numbers of sterile males is within reach. This success provides opportunity to revive the SIT component of the program to eradicate *G. pallidipes* from the fertile Rift Valley lands of Ethiopia [182]. This would translate into availability of more animals for plowing the fertile land, more milk, and manures to plant crops—in short, a permanent eradication of poverty and improvement of living standards. Similarly, other sub-Sahara African countries infested with trypanosome-transmitting tsetse species would benefit from this success. It should be noted that, although GpSGHV has not been shown to cause serious problems in other tsetse species, the virus infects *G. fuscipes fuscipes*, *G. morsitans* and *G. swynnertoni*, irrespective of their ages, sex, and season of the year [183]. Therefore, if GpSGHV (or a similar virus) will be problematic in the future, the research reviewed in this paper provides a solid basis to deal with the problem. The current knowledge and experiences in large-scale production of *G. pallidipes* can be used to make recommendations on standard operational procedures (SOPs) for the management of GpSGHV in large-scale tsetse fly production facilities. The proposed SOP is detailed in Supplementary File 1.

## 9. Concluding Remarks

The aim of this review was to highlight the intricacies associated with the occurrence of a newly described group of viruses, the hytrosaviruses, and the interaction with their dipteran hosts. The common denominators are the structure and genome content of these viruses and their potential to induce the SGH syndrome. Despite the knowledge of the genome and proteome, very little is known about the regulation of viral gene expression and the interaction of viral proteins with the dipteran host and its microbiome and parasites. Nevertheless, the wealth of information available in a relatively short period through the concerted efforts of many research groups [184] allowed the mitigation and control of SGH in tsetse fly colonies and set the stage for the development of more advanced strategies for tsetse fly control to eliminate trypanosomosis. A future challenge for virologists is to understand the natural role of hytrosaviruses in dipteran fly ecology and evolution.

## Supplementary Files

Supplementary File 1
