# Supplementary material for: Virology, Epidemiology and Pathology of Glossina Hytrosavirus, and Its Control Prospects in Laboratory Colonies of the Tsetse Fly, Glossina pallidipes (Diptera; Glossinidae)
Source: Insects. 2013 Jul 2;4(3):287–319. doi: 10.3390/insects4030287 (PMC4553466; doi:10.3390/insects4030287)
Supplement: Supplementary File 1 [file insects-04-00287-s001.doc]

*Insects* **2013**, *4*, 1-x manuscripts; doi:10.3390/insects40x000x

§

**OPEN ACCESS**

***insects***

**ISSN 2075-4450**

www.mdpi.com/journal/insects/

Review

Virology, Epidemiology and Pathology of *Glossina* Hytrosavirus, and Its Control Prospects in Laboratory Colonies of the Tsetse Fly, *Glossina pallidipes* (Diptera; Glossinidae)

Henry M. Kariithi ^1,2,3^,*, Monique M. van Oers ^1^, Just M. Vlak ^1^, Marc J. B. Vreysen ^2^,
Andrew G. Parker ^2^ and Adly M. M. Abd-Alla ^2^

^1^ Laboratory of Virology, Wageningen University, Droevendaalsesteeg 1, Wageningen 6708 PB,
The Netherlands; E-Mails: monique.vanoers@wur.nl (M.M.O.); just.vlak@wur.nl (J.M.V.)

^2^ Insect Pest Control Laboratories, Joint FAO/IAEA Programme of Nuclear Techniques in Food and Agriculture, International Atomic Energy Agency, Wagrammer Strasse 5, P.O. Box 100, 1400 Vienna, Austria; E-Mails: m.vreysen@iaea.org (M.J.B.V.); a.g.parker@iaea.org (A.G.P.); a.m.m.abd-alla@iaea.org (A.M.M.A.-A.)

^3^ Biotechnology Centre, Kenya Agricultural Research Institute, Waiyaki Way;
P.O. Box 14733-00100, Nairobi, Kenya

***** Author to whom correspondence should be addressed; E-Mail: henry.kariithi@wur.nl;
Tel.: +31-317-483-099.

The following standard operational procedures are recommended to manage infections of the *Glossina pallidipes* salivary gland hypertrophy virus (GpSGHV) in the laboratory colonies of the tsetse flies. The SOPs were developed based on the current knowledge and experiences in large-scale production of *G pallidipes*.

Standard Operational Procedures to Manage GpSGHV in Tsetse Fly 'Factories'

The current knowledge and experiences in large-scale production of *G pallidipes* can be used to make recommendation on standard operational procedures on how to manage GpSGHV in large-scale tsetse fly production facilities.

1. Staff involved in tsetse rearing must be sufficiently educated to recognize SGH symptoms, and to regularly monitor variations from normal “healthy” flies. Such variations include reproductive disturbances (reduced matings and egg production) and longevity (premature mortalities and prolonged larviposition cycles).
2. To establish new tsetse colonies from field-collected immature flies and/or pupae, it is recommended to use of teneral flies produced by SGH-free mothers. Therefore, pregnant females, and the pupae produced by these mothers should be kept separately and, if possible, individually. The mothers should then be dissected to assess SGH symptoms. Where possible, the virus infection status of these mothers should be confirmed by PCR. Only progenies produced by SGH-free mothers should be used as seed for the new tsetse colonies.
3. When colonies are initiated from tsetse flies or pupae obtained from other tsetse rearing facilities, it is recommended that it should be enquired from the facility manager about the 'health' status of their colony(s), and whether the insects have been screened for SGH. If the colony has not been screened, the new insects should be quarantined and screened for viral infections before establishing them in the SGH-free colony. It is further recommended that the rearer from who the fly or pupae were obtained should be informed on the findings of the screening efforts.
4. To suppress the asymptomatic infections in teneral flies (young colony units), blood meals should be supplemented with the antiviral drug, valacyclovir (to block viral DNA replication) at a maximum of 300 µg per ml of blood. If valacyclovir is unavailable, it is possible to start a clean feeding system on the non-supplemented blood. Since valacyclovir is a relatively cheap antiviral drug, it is recommended that blood meals should be supplemented with the drug for the colonies in the entire tsetse facilities. Alternate application of antiviral drugs at regular intervals (to prevent development of resistance to the drug) will significantly reduce the virus load in the colony and therefore prevent development of SGH. The cost is expected to reduce over time because the drug is more effective with lower viral loads, and can then be used at the lower doses.
5. Fly colonies should be maintained on a clean feeding regime (one feeding round per membrane) as much as possible. When the numbers of flies in such a colony reach the maximum number that can be handled in one feeding round, the surplus flies can be used to initiate a new colony. This second colony should be maintained on a second feeding round after feeding the first colony, and the cycle can be used to initiate other successive colonies. Importantly, the pupae produced by each of the colonies should be collected and incubated separately from the regular colonies, and the records for each of the colonies should be kept separately.
6. Excess male flies should be sampled weekly and dissected to monitor the prevalence of SGH symptoms recorded. SGH prevalence rates of ≤10% are acceptable. SGH prevalence ≥10% should be considered as an indicator of a colony under risk.
7. For the tsetse colonies that are already established, at least 20 flies (males and females) should be sampled from each weekly unit and dissected to assess SGH prevalence. In case the SGH prevalence is ≥10%, blood meals offered to the colonies should be immediately be supplemented with valacyclovir. Additionally, the clean feeding regime should be implemented.
8. Since implementation of the clean feeding system does not incur additional materials and rearing staff, it is recommended to use this regime even when SGH is assumed to be eradicated from the colonies.

© 2013 by the authors; licensee MDPI, Basel, Switzerland. This article is an open access article distributed under the terms and conditions of the Creative Commons Attribution license (http://creativecommons.org/licenses/by/3.0/).
